# Therapeutic Applications of *Aggregatibacter actinomycetemcomitans* Leukotoxin

**DOI:** 10.3390/pathogens13050354

**Published:** 2024-04-25

**Authors:** Scott C. Kachlany, Brian A. Vega

**Affiliations:** Department of Oral Biology, Rutgers School of Dental Medicine, Newark, NJ 07103, USA

**Keywords:** LFA-1, inflammation, leukemia, lymphoma, autoimmune disease, translational research, drug development, protein drug, allergic asthma, inflammatory bowel disease, Crohn’s disease, ulcerative colitis

## Abstract

*Aggregatibacter actinomycetemcomitans* is a Gram-negative oral bacterium that has been primarily studied for its role in causing periodontal disease. The bacterium has also been implicated in several systemic diseases such as endocarditis and soft tissue abscesses. Leukotoxin (LtxA) is perhaps the best studied protein virulence factor from *A. actinomycetemcomitans*. The protein can rapidly destroy white blood cells (WBCs), helping the bacterium to subvert the host immune system. The functional receptor for LtxA is lymphocyte function associated antigen-1 (LFA-1), which is expressed exclusively on the surfaces of WBCs. Bacterial expression and secretion of the protein are highly regulated and controlled by a number of genetic and environmental factors. The mechanism of LtxA action on WBCs varies depending on the type of cell that is being killed, and the protein has been shown to activate numerous cell death pathways in susceptible cells. In addition to serving as an important virulence factor for the bacterium, because of its exquisite specificity and rapid activity, LtxA is also being investigated as a therapeutic agent that may be used to treat diseases such as hematological malignancies and autoimmune/inflammatory diseases. It is our hope that this review will inspire an increased intensity of research related to LtxA and its effect on Aggressive Periodontitis, the disease that led to its initial discovery.

## 1. Introduction

*Aggregatibacter actinomycetemcomitans* produces numerous well-studied virulence factors including cytolethal distending toxin, tight adherence pili, cell-specific adhesins, and leukotoxin (LtxA). Leukotoxin from *A. actinomycetemcomitans* has been studied primarily as a bacterial virulence factor that contributes to the disease process. The protein is thought to help the bacterium to evade the host immune response during infection by eliminating certain white blood cells. The clinical relevance of LtxA is supported by the fact that patients who harbor the highly leukotoxic JP2 strain have an increased risk of developing aggressive periodontal disease [1]. Because of its numerous unique properties, our laboratory has also been studying how the protein can serve as a potent and safe therapeutic agent. Much like other bacterial proteins, such as botulinum toxin and *E. coli* asparaginase, which have been used as effective FDA-approved drugs, leukotoxin (LtxA) has significant potential to treat patients with various white blood cell (WBC) disorders, such as hematologic malignancies and autoimmune/inflammatory diseases.

The seminal discovery by Lally et al. [2,3] that LtxA binds specifically to lymphocyte function associated antigen-1 (LFA-1) laid the foundation for understanding how the protein interacts with host cells and, subsequently, how LtxA can be used to treat patients with disease. LFA-1 is a beta-2 integrin that is a dimer between CD11a and CD18 [4,5,6]. LFA-1 is surface localized and found exclusively on WBCs, which explains the target specificity of the protein. LFA-1 is highly conserved across species and considered essential for maintenance of a functional immune system. Initially, there was a controversy as to how LtxA was delivered [7,8,9,10]. Research suggested that LtxA was delivered in extracellular blebs and not secreted; however, it was subsequently discovered that LtxA was found in both secreted [11,12] and extracellular blebs [13,14,15], providing the toxin with both a direct attack on cell membranes (in the secreted form) and an intracellular attack mode (by blebs fusing with outer cell membranes).

The physiological role of LFA-1 in mammals is to interact with intercellular adhesion molecule-1 (ICAM-1) on vascular endothelial cells and mediate the proliferation and migration of the WBC from the blood stream into the surrounding tissue [6,16,17]. This migration occurs when WBCs are needed at certain locations in the body due to infection or injury. Inflammatory cytokines, such as IL-6 and IL-8, serve as signals to mediate the interaction between LFA-1 and ICAM-1 and subsequent trans-endothelial transmigration. However, not all WBCs in the body interact with ICAM-1 constantly. In fact, most WBCs exist in a resting state, whereby they are merely surveying the environment, waiting to be called upon by the immune system. The interaction between LFA-1 and ICAM-1 is controlled by the activation state of LFA-1. LFA-1 can exist in three different conformations: a low-, intermediate-, and high-affinity state [18,19,20,21]. In the low-affinity conformation, the CD11a and CD18 molecules are closed in a tucked-in structure that makes them unavailable to bind to ICAM-1. This is the resting state that the majority of circulating and tissue-resident WBCs exhibit. In the intermediate state, LFA-1 is extended and can interact with ICAM-1, albeit in a low-affinity capacity. In the high-affinity conformation, LFA-1 is extended and its ligand-binding site is fully available, allowing maximal interaction with ICAM-1. The activation state of LFA-1 is regulated by chemicals (such as PMA), metals (such as Mn^+^, Mg^2+^, and Ca^2+^), and cytokines.

While interaction between LFA-1 and ICAM-1 under physiological conditions mediates the proliferation and migration of WBCs, contact between LFA-1 and LtxA results in very rapid cell death. Interestingly, it was discovered that LtxA preferentially kills activated WBCs that express the high-affinity LFA-1 conformation [22,23]. Physiologically, this preference makes the most sense since activated, proinflammatory WBCs pose the greatest threat to the bacterium. Hence, LtxA naturally targets the most active components of the immune system.

Studies on the WBC killing mechanisms by LtxA have revealed numerous fascinating and novel discoveries. There are significant differences in how LtxA kills macrophages and monocytes compared to lymphocytes. While these mechanisms have been described extensively elsewhere [24,25,26,27,28,29], a brief summary is described below. For macrophages and monocytes, LtxA interaction with active LFA-1 results in at least two downstream events: the activation of a caspase apoptotic pathway and endocytosis of the LtxA-LFA-1 complex and delivery to the lysosome. LtxA appears to cause significant disruption and damage to the lysosomal membrane, which seals the fate of the cell. Thus, it appears that LtxA activates at least two non-redundant killing pathways to ensure death of the cell.

For T-lymphocytes, LtxA initially contacts LFA-1 on the surface and then also appears to recruit FAS (CD95) death receptor to the complex to activate the FAS death receptor pathway. Normally, FAS death receptor requires contact with FAS ligand (FASL) for the activation and initiation of cell death. However, LtxA bypasses the requirement for FASL and is able to directly activate caspase-8 and the induction of cell death. It is interesting to note that macrophages and monocytes express very little FAS receptor on their surface, while lymphocytes are known to express abundant levels. Furthermore, macrophages and monocytes have a high lysosomal content, while lymphocytic cells contain a relatively small number of lysosomes. Hence, LtxA may have evolved numerous mechanisms to kill cells based on the death pathways available in each cell type.

While LtxA targets specifically cells that express LFA-1, not all cell types are equally sensitive to the bacterial protein. Both the surface levels of LFA-1 and activation state of the molecule affect sensitivity to LtxA. In general, neutrophils and monocytes have the highest expression levels of LFA-1, followed by T cells and then B cells. Indeed, the sensitivity of these cell types is roughly proportional to the levels of LFA-1, with monocytes and neutrophils being killed most efficiently by LtxA. In vitro and ex vivo studies have also shown that WBCs that are activated with molecules such as phorbol esters or certain activating antibodies exhibit 10–100 times greater sensitivity to LtxA than non-activated cells [22,23,24]. Furthermore, in vivo studies in rats and dogs have demonstrated that intravenously administered LtxA preferentially targets cells with the highest levels of active LFA-1.

A large number of white blood cell diseases are characterized by the overexpression and activation of LFA-1 such as certain hematologic malignancies and autoimmune/inflammatory diseases, including inflammatory bowel disease (Crohn’s disease and ulcerative colitis), rheumatoid arthritis, psoriasis, multiple sclerosis, type I diabetes, allergic asthma, and dry-eye disease [30,31,32,33,34,35,36,37]. Given LtxA’s preferential killing of cells expressing high levels of active LFA-1, we postulated that LtxA may serve as an ideal therapeutic agent for specifically and safely targeting diseased WBCs. This preferential attack on distinct cell populations is in need of further exploration in periodontal diseases. Below is a description of the multitudinous ways in which LtxA can be used for treating diseases other than periodontitis. This broad spectrum of effects supports the concept that LtxA can effect host immune regulation in several divergent ways.

## 2. Hematologic Malignancies

Leukemia and lymphoma are cancers of the immune system that are often difficult to treat [38,39,40,41]. The 5-year survival rate for patients with acute myeloid leukemia (AML) is 30%, which decreases with increasing age. Furthermore, nearly 50% of AML patients relapse after successful treatment. B cells and T cells can also become malignant and may circulate throughout the body or grow within lymph nodes. Because LtxA is able to potentially kill different subsets of WBCs selectively, we initially proposed that LtxA may be an ideal (effective and targeted) therapeutic agent to treat patients with leukemia and lymphoma. In these cases, the goal is merely to wipe out the cancerous WBCs.

We have evaluated LtxA in numerous animal models for leukemia and lymphoma [23,27]. In each study, just a few doses of LtxA were sufficient to eliminate the cancer, and the cancer never returned, suggesting that cells did not develop resistance to the protein. Interestingly, we never observed resistance to LtxA in any of the animal studies or in vitro studies using cancer cell lines derived from leukemia and lymphoma patients. Because LtxA employs numerous mechanisms to kill a cell, we believe that it would be difficult for a cell to develop resistance to the protein. This property could make LtxA a very effective therapy with a high unmet need.

**Figure 1:**
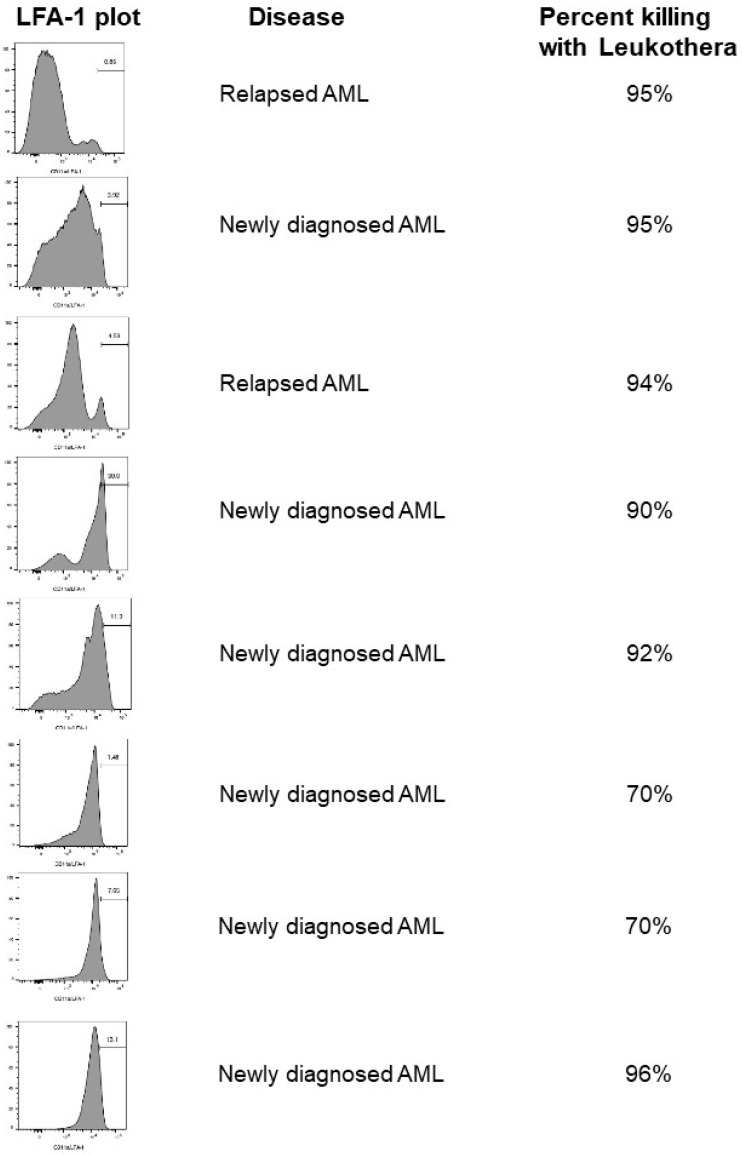
LFA-1 on peripheral blood mononuclear cells (PBMCs) from AML patients. PBMC samples from patients with AML were stained with anti-CD11a antibodies and then analyzed via flow cytometry. The *x*-axis represents the levels of LFA-1, and the y-axis represents intensity of the peak. Cell viability was also measured in cells that had been separately treated with LtxA for 4 h. Viability was determined using the ATP-based Cell-Titer Glo Assay. Leukothera is a potential commercial name for LtxA.

We have also evaluated the levels of LFA-1 on WBCs from patients with various hematologic malignancies, including AML (Figure 1). We found that the levels of LFA-1 varied from low to very high levels on both newly diagnosed and relapsed AML patient samples. Furthermore, regardless of the levels of LFA-1, LtxA had a significant effect on the cells, suggesting that even low levels of LFA-1 are sufficient for activity (Figure 1). It may also be that these cells express the highly active conformation of LFA-1, making them even more sensitive to LtxA.

In 2021, a pharmaceutical company (Actinobac Biomed, Inc., manufactured in Baltimore, MD, USA) received IND approval from the FDA to evaluate intravenous LtxA in patients with relapsed/refractory leukemia and lymphoma, and these trials are pending. 

## 3. Psoriasis

Psoriasis is the most common autoimmune disease, affecting about 2–3% of the population [42,43,44,45]. The disease presents in the form of dry, scaley skin that causes significant discomfort and itchiness for patients. The dry, patchy areas of skin, known as psoriatic plaques, are the result of keratinocytes proliferating and migrating to the surface of the skin much faster than normal, where they accumulate as a mass of cells. This enhanced proliferation and migration of skin cells is caused by proinflammatory cytokines that are secreted by over-reactive WBCs (predominantly T cells) in the underlying vasculature. Strategies to treat psoriasis include therapeutics directed against these cytokines, as well as drugs that suppress the immune system (such as steroids).

Given that LFA-1 plays a crucial role in the migration of T cells to the affected tissue in patients with psoriasis, we postulated that LtxA could be an effective strategy to eliminate the highly reactive immune cells without affecting the healthy, resting WBCs. We tested this hypothesis using a humanized mouse model for psoriasis [46]. In brief, human skin from patients with severe plaque psoriasis was grafted onto the backs of mice, and then the mice were treated with LtxA. In contrast to the vehicle control, LtxA treatment was able to essentially reverse the established disease and restore all parameters of healthy skin, including dermal and epidermal thickness, lymphocyte infiltration, and clinical psoriasis score (the visual appearance of the skin).

Further evidence that LtxA could be effective for treating inflammatory skin conditions was provided in a canine model for atopic dermatitis (AD), a common skin condition in dogs that results in extensive scratching and hair loss. Dogs with AD that were treated with a single dose of LtxA exhibited skin that was pathologically returned to health (unpublished results).

## 4. Allergic Asthma

Allergic asthma is a condition that is often activated by allergens such as house dust mite and pollen. The result is an accumulation of inflammatory WBCs, such as neutrophils and macrophages, in the lung tissue and the production of proinflammatory cytokines [47,48,49,50,51]. Since LFA-1 plays a significant role in the migration of WBCs to the lung tissue, we sought to determine if WBCs from patients with allergic asthma had higher levels of LFA-1 than those of healthy controls, as well as if LtxA could be effective in a mouse model for the disease [52]. 

Patients with allergic asthma exhibited WBCs that expressed significantly higher levels of LFA than those of controls. Most strikingly, these patients harbored a subpopulation of LFA-1-high, CD4-negative WBCs that was completely absent from healthy control subjects. It is possible that this unique subpopulation of cells is responsible for the over-reactive immune response in patients with allergic asthma. Furthermore, ex vivo treatment of WBCs from patients with LtxA revealed that LtxA preferentially eliminated the WBCs expressing the highest level of LFA-1, while not affecting the healthy, resting-state cells.

We evaluated LtxA in a mouse model for allergic asthma. Mice that are treated with house dust mite extract through inhalation develop pulmonary inflammation and a pathology that resembles the human condition. Most notably, a significant infiltrate of WBCs is detected in the lung sections of the mice. In the employed model, house dust mite extract treatment is administered for several weeks to allow the development of the disease. Systemic LtxA was then given to mice, and the treatment was compared to vehicle control. Mice that were treated with LtxA exhibited a significant reversal of disease based on WBC infiltrate levels in lung tissue and bronchoalveolar lavage (BAL) fluid and inflammatory cytokines in the BAL fluid. Interestingly, the WBCs in the BAL fluid expressed approximately 10-fold higher levels of LFA-1 than the WBCs in the peripheral blood, suggesting that cells migrating to the airways require LFA-1 to localize there.

## 5. Inflammatory Bowel Disease

Inflammatory bowel diseases (IBDs) such as Crohn’s disease (CD) and ulcerative colitis are the result of an over-reactive immune system that targets the gastrointestinal tissue, resulting in inflammation and subsequent abdominal pain, bleeding, weight loss, and malnutrition [53,54,55,56,57]. Several studies have shown that LFA-1 plays a crucial role in the development of the disease. 

Animal and human studies have shown that LFA-1 plays a critical role in the induction and progression of IBD. Multiple studies using the adoptive T-cell transfer model of chronic colitis indicated that the transfer of LFA-1 deficient T cells failed to induce chronic colitis in RAG-1^−/−^ recipient mice, whereas the transfer of wild-type T cells induced severe colitis in the same immunodeficient recipients. Failure to induce colonic inflammation correlated with a significant reduction in CD3^+^CD4^+^ T cell numbers within the mesenteric lymph nodes (MLNs) and colonic interstitium. These data demonstrate that LFA-1 is critical for migration and/or priming within the MLN and the subsequent recruitment of these T cells into the colonic interstitium to initiate disease [58,59]. Additionally, in a dextran sodium sulfate (DSS)-induced model of colitis, the loss of LFA-1 significantly attenuated the development of disease by decreasing leukocyte infiltration and subsequent tissue damage in the intestine [60]. Furthermore, in a small open-label study with Efalizumab (anti-LFA-1 antibody), it was found that after 8 weeks of treatment, 67% of patients with moderate-to-severe refractory CD had a clinical response and 40% went into remission [61]. Collectively, these studies validate LFA-1 as a target in IBD.

**Figure 2:**
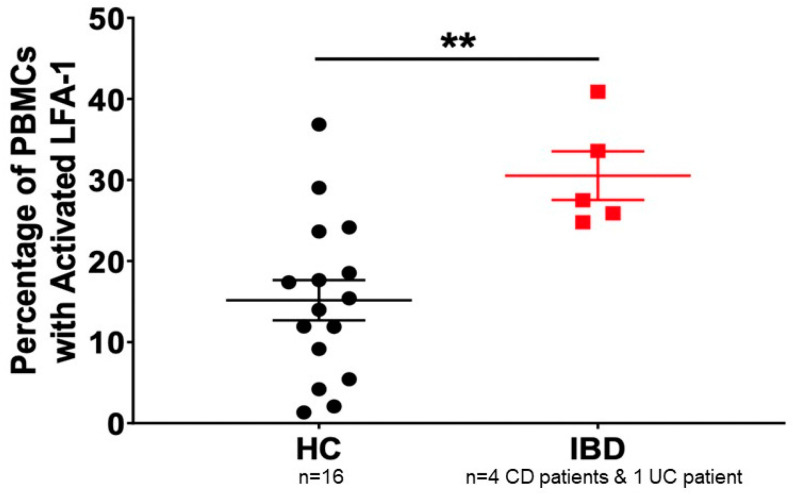
LFA-1 levels in PBMCs derived from healthy controls and patients with IBD. PBMCs were isolated and analyzed using anti-LFA-1 antibody and analyzed via flow cytometry. HC = healthy control; IBD = patients with Crohn’s disease (CD) or ulcerative colitis (UC). ** indicates *p* ≤ 0.05.

**Figure 3:**
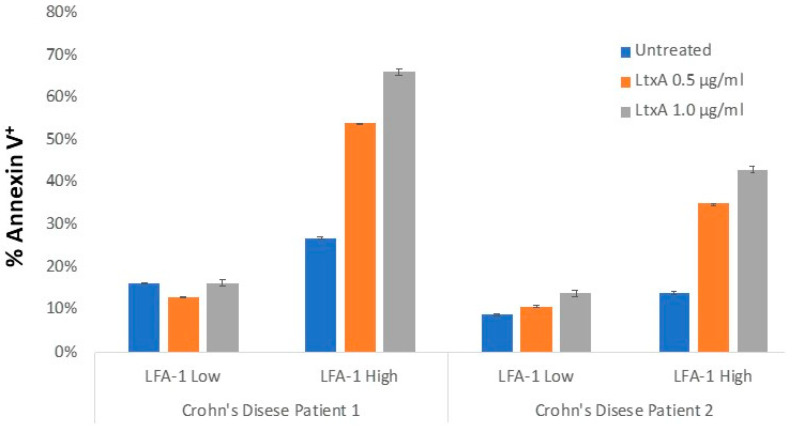
Sensitivity of PBMCs derived from CD patients to LtxA. PBMCs from two CD patients were treated with LtxA, and then cells were stained with anti-LFA-1 antibody (to separate high and low populations) and Annexin-V (to detect cell death).

We have conducted additional studies with the goal of assessing whether leukocytes derived from patients with IBD express a higher percentage of LFA-1 compared to healthy controls and whether these cells are sensitive to LtxA. We isolated PBMCs derived from healthy controls and IBD patients and assessed the percentage of PBMCs that express LFA-1 via flow cytometry (Figure 2). We found that PBMCs derived from IBD patients expressed significantly more LFA-1 compared to healthy controls (Figure 3). Furthermore, these highly expressing LFA-1 cells were sensitive to LtxA. 

**Figure 4:**
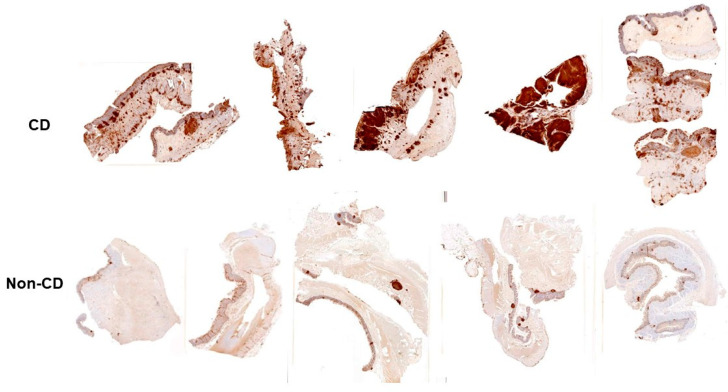

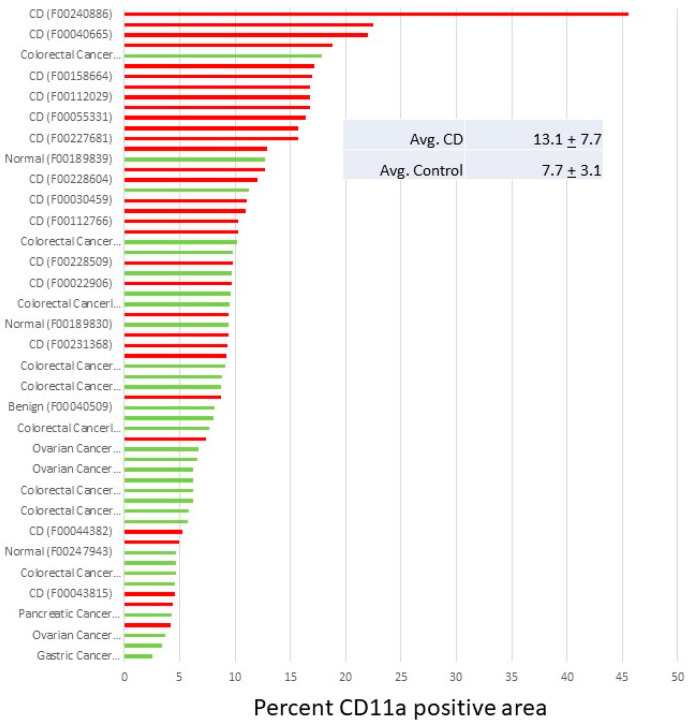
Immunohistochemical staining of LFA-1 on GI tissue. (**Top**) Intestinal tissue from CD patients or non-CD individuals were stained with anti-CD11a antibody and processed using immunohistochemistry. Shown are representative histological samples. (**Bottom**) The intensity of LFA-1 staining in the tissue samples was quantified as the percentage positive staining area compared to the total tissue area. Red bars represent CD samples, while green bars represent non-CD samples.

We also carried out a study of intestinal tissue to determine if WBCs in the tissue sourced from Crohn’s disease patients expressed higher levels of LFA-1 than those from non-CD patients. We found that LFA-1 staining in tissue from CD patients was significantly greater than similar tissue from non-CD patients (Figure 4). Thus, LtxA may be an effective approach to eliminate highly reactive immune cells in the GI tracts of patients suffering from IBD.

## 6. Conclusions

It is clear from the above review that LtxA has many ways of influencing the host response and affecting disease outcomes. In this review, we have highlighted the utility of LtxA as a therapeutic agent by modulating host immune responsiveness in a broad spectrum of immune cell diseases. It is our hope that showing this diverse efficacy can reinforce the idea that LtxA plays an important role in the disease of interest in this volume, namely periodontal disease.
